# Microfluidic system for monitoring temporal variations of hemorheological properties and platelet adhesion in LPS-injected rats

**DOI:** 10.1038/s41598-017-01985-w

**Published:** 2017-05-11

**Authors:** Eunseop Yeom, Hye Mi Kim, Jun Hong Park, Woorak Choi, Junsang Doh, Sang Joon Lee

**Affiliations:** 1 School of Mechanical Engineering, Pusan National University, Busan, South Korea; 2 Division of Integrative Biosciences and Biotechnology (IBB), Pohang University of Science and Technology (POSTECH), Pohang, South Korea; 3 Department of Mechanical Engineering, Pohang University of Science and Technology (POSTECH), Pohang, South Korea

## Abstract

Sepsis causes multiple organs failures and eventually death. Changes in blood constituents due to sepsis lead to alterations in hemorheological properties, and cell adhesiveness. In this study, a new microfluidic system is proposed to measure temporal variations in biophysical properties of blood after injecting lipopolysaccharide (LPS) into a rat extracorporeal model under *ex vivo* condition. To measure blood viscosity, the interfacial line between blood and a reference fluid is formed in a Y-shaped channel. Based on the relation between interfacial width and pressure ratio, the temporal variation in blood viscosity is estimated. Optical images of blood flows are analyzed by decreasing flow rate for examination of red blood cell (RBC) aggregation. Platelets initiated by shear acceleration around the stenosis adhere to the post-stenosed region. By applying a correlation map that visualizes the decorrelation of the streaming blood flow, the area of adhered platelets can be quantitatively attained without labeling of platelets. To assess sepsis inflammation, conventional biomarkers (PCT and IL-8) are also monitored. The increasing tendency for blood viscosity, RBC aggregation, platelet adhesion, and septic biomarkers are observed after LPS injection. This microfluidic system would be beneficial for monitoring the changes in hemorheological properties and platelet activation caused by sepsis.

## Introduction

Sepsis is the most frequent cause of mortality in numerous intensive care units^1^. This complex and impetuous illness induces alterations in microcirculation and changes in biochemical and physiological characteristics of blood constituents^2^. Symptomatically, this pathophysiological processes are manifested by two or more of the following symptoms: fever or hypothermia, tachypnea, tachycardia, leukocytosis or leukopenia^3^. In particular, microvascular damages caused by sepsis play a crucial role in the impairment of tissue oxygenation, resulting in multiple organ failure and eventually death^4^.

Therefore, diagnosis and severity stratification of sepsis are essential for timely and specific clinical treatment^5^. Considering that useful biomarkers function as suitable therapeutic guides to indicate the presence, absence, or severity of sepsis, adequate biomarkers are necessary for precise diagnosis of sepsis and accurate evaluation of its severity. In order to identify the bacterial etiology of sepsis, biomarkers including procalcitonin (PCT), C-reactive protein (CRP), interleukin (IL)-6 and IL-8 have been extensively used^3, 6, 7^.

Alterations in blood constituents in patients result in changes in viscosity and rheological features of red blood cells (RBCs) and white blood cells (WBCs)^8, 9^. Sepsis in animals and patients decreases RBC deformability and increases aggregation and adhesion among WBCs, platelets, and endothelial cells^10, 11^. Microcirculatory dysfunctions may be further aggravated by such hemorheological changes^12^. Therefore, detailed information on hemorheological properties can provide novel perspectives on the diagnosis of sepsis^13^.

Besides hemorheological properties, platelets can either stimulate or inhibit regulatory functions of other cellular partners in the inflammatory process. Specifically, activated platelets release or produce many small molecules such as RANTES, IL1-β, monocyte chemoattactant factor (MCP-1), platelet factor 4 (PF4), and platelet activating factor (PAF), to regulate inflammation and tissue repair processes in patients with sepsis^14^. These molecules may promote platelet–immune cell adhesion^15^. The activation of platelets, the coagulation system, and inflammatory processes are involved in a detrimental interplay and thrombocytopenia^16^. Given that profound thrombocytopenia is observed in severe sepsis, platelet function may be associated to some extent with the severity of sepsis^17^. Therefore, the readouts of platelet activation have been suggested as biomarkers to indicate the infectious state of sepsis complications and the related prognosis^14, 16^.

The erythrocyte sedimentation rate (ESR) has been widely used clinically as an indicator of inflammation in the diagnosis of chronic diseases^18, 19^. An ESR value reflects certain hemorheological conditions, such as RBC aggregation, RBC deformability, and hematocrit^20^. However, measurement of ESR values is a tedious process. Moreover, other assessment techniques (rotational viscometer, capillary viscometers, light scattering analysis, and ultrasonic analysis) used to measure hemorheological properties require a large amount of blood samples for repetitive tests^21–23^. Compared with conventional measurement tools, microfluidic devices have distinctive advantages including such as small sample volume and high sensitivity^24, 25^. In our previous studies, several microfluidic techniques were proposed to measure various hemorheological properties, including RBC aggregation, blood viscosity, and platelet adhesion, based on the optical images of blood samples in microfluidic devices^20, 26, 27^.

Considering that *ex vivo* measurements can provide reliable information, several microfluidic devices have been recently adopted for *ex vivo* monitoring of temporal variations in biophysical properties of blood samples^28–30^. By inserting these microfluidic devices into a rat extracorporeal model in which blood circulates through an external loop that directly connects both the artery and vein, a number of biophysical properties can be measured as a function of time under *ex vivo* conditions^31–33^. Since animal models of sepsis with systemic clinical signs (fever, lethargy, shivering, and piloerection) can be made by injecting stimulatory agents such as lipopolysaccharide (LPS) or endotoxin^34^, a extracorporeal model with sepsis can be established through a single injection of LPS^28^. In this study, we propose a microfluidic system for monitoring temporal variations in biophysical properties including blood viscosity, RBC aggregation, and platelet adhesion, in the rat extracorporeal sepsis model after injecting LPS. To assess inflammation caused by sepsis, conventional biomarkers of PCT and IL-8 are also monitored.

## Microfluidic system for ***ex vivo*** monitoring

**Figure 1:**
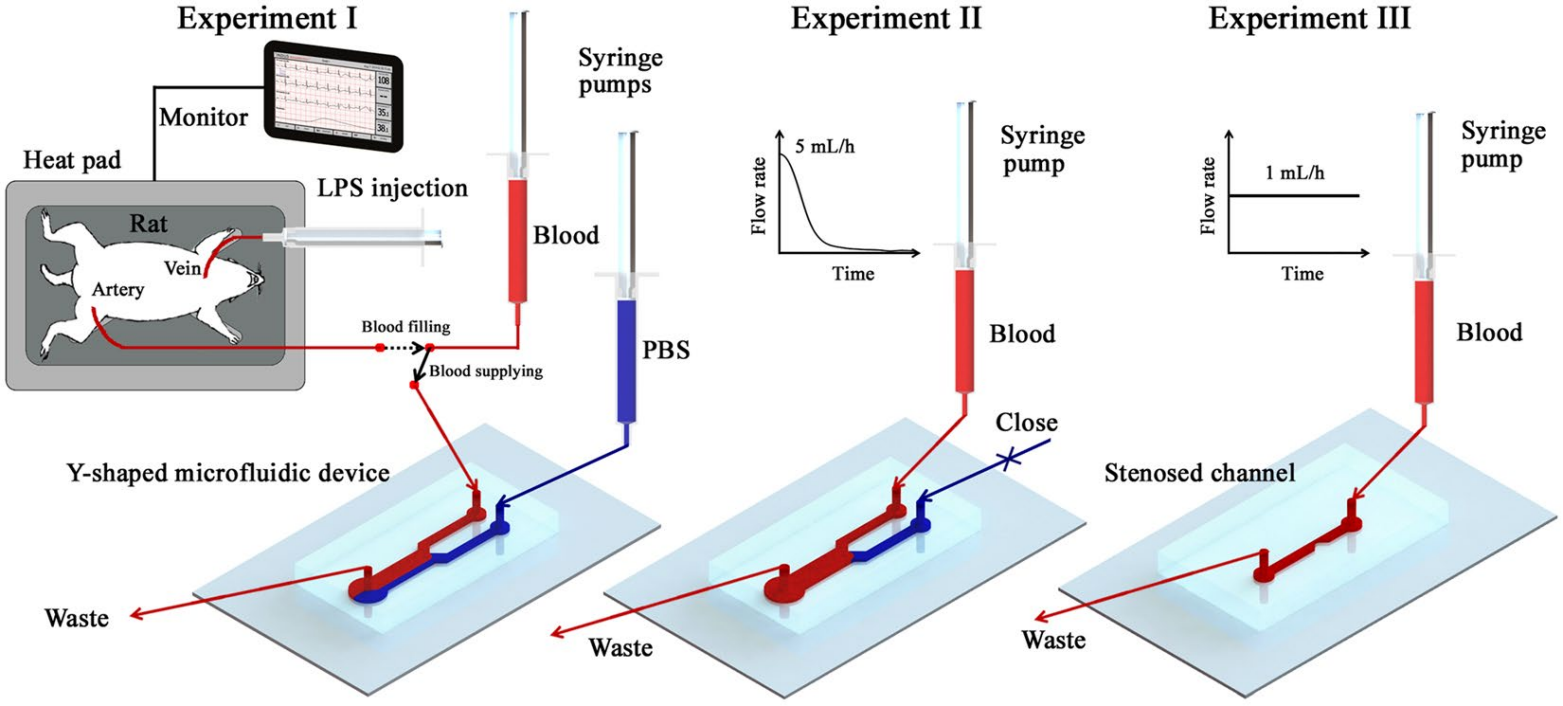
Measurement procedure of the microfluidic system for monitoring blood viscosity, RBC aggregation and platelet adhesion. A rat extracorporeal conduit is established by connecting the artery of a rat and a microfluidic conduit. By manipulating a three-way valve, blood in the rat artery is filled in the syringe and supplied into the microfluidic devices. Viscosity of blood sample is measured by inducing an interfacial line between a reference PBS solution and test blood sample in the Y-shaped microfluidic device (Experiment I). After measuring viscosity variation according to shear rate, Experiment II is carried out to measure RBC aggregation while the inlet of PBS solution is closed. Flow rate of test blood sample is reduced from 5 mL/h to 0 mL/h to induce the formation of RBC aggregates. For the estimation of platelet adhesion, Experiment III is performed by connecting a microfluidic channel with stenosis (90% severity) into the rat extracorporeal conduit. Flow rate of the blood sample is maintained at 1 mL/h during Experiment III.

Hemorheological properties are affected by external exposure of blood samples^35^, so *ex vivo* measurements can accurately estimate these properties without remarkable changes. The rat extracorporeal model has been demonstrated to monitor temporal variations in hemorheological and hemodynamic properties with high accuracy under *ex vivo* conditions^32, 33^. A digital image processing technique has been recently introduced for accurate estimation of platelet adhesion^27^. Specifically, a correlation map labels the 2D correlation coefficient (*R*) in the small tiles of two consecutive images captured with a time interval Δ*t*. Given that the *R* values of adhered platelets are high, the area for adhered platelets is distinguished from flowing blood with low *R* values. In order to monitor temporal variations in biophysical properties after LPS treatment, three experiments are carried out using two different microfluidic devices (Fig. 1).

In the rat extracorporeal conduit, an artery of the rat model is connected to an empty syringe in the pump. By withdrawing the syringe piston, 2.5 mL of blood in the rat artery is filled in the empty syringe. A three-way valve is placed in the extracorporeal conduit to adjust flow direction. By manipulating the three-way valve, rat blood is supplied into the microfluidic devices without external exposure of blood samples. To maintain rat physiological condition during sepsis treatment, the body temperature is maintained by using a heat pad that monitors body temperature. As a result, any noticeable variation in body temperature is not observed for all test samples (Table 1).

**Table 1:** Temporal variation of temperature and hematocrit after LPS injection.

	0 h	1 h	3 h	6 h
Body temperature (°C)	34.18 ± 0.59	35.13 ± 0.15	36.94 ± 0.39	35.20 ± 0.32
Hematocrit	47.86 ± 0.82	45.76 ± 0.93	48.28 ± 0.61	50.38 ± 1.14

Each value represents mean ± standard error of the mean.

In Experiment I, PBS solution and blood sample are delivered into the Y-shaped microfluidic device through each inlet to generate an interfacial line between them. Given that the interfacial line between two different flows is related to the pressure ratio between them^36^, the bulk viscosity of blood flow can be estimated by analyzing the width ratio of the interfacial line. After measuring blood viscosity, Experiment II is carried out using the same Y-shaped microchannel for measuring RBC aggregation. To prevent unwanted flow, the inlet of PBS solution is tightly closed by the clip. To induce RBC aggregation, the flow rate of a blood sample is reduced from 5 mL/h to 0 mL/h^20^. The formation of RBC aggregates creates clear speckle patterns in the optical images of blood flows. The extent of RBC aggregation can be reasonably estimated trough image analysis.

In Experiment III, a microfluidic channel with a stenosis is employed to accentuate platelet activation. Blood is stably supplied into the stenosed microchannel at a flow rate of 1 mL/h. To accurately estimate the area of adhered platelets in flowing blood, the captured optical images are analyzed by using digital image processing techniques (masking, correlation mapping, and thresholding). After Experiment III, the hematocrit and concentrations of PCT and IL-8 are measured by using the blood sample remaining in the syringe pump. Sepsis is induced by injecting LPS to the rat vein. After the lapse of a specific time (1, 3, and 6 h), the same procedure is repeated.

Similar to the present method, a previous method using H-shaped microchannel estimated blood viscosity by measuring an interfacial line between the test sample and the PBS solution, and the adhesion of platelets through digital image processing techniques^27^. The interfacial line is induced by blocking the outlet of one side-channel in H-shaped channel. This blockage easily makes air bubbles trapped in the blocked channel. The transient response time can be changed due to compressibility of air trapped in the device as the flow rate varies. In addition, it is difficult to measure adhesion of platelets in the upstream of H-shaped channel because the hyper-adhesion of platelets induced by LPS treatment can easily block the channel. To address these problems, variations of blood viscosity and platelet adhesion are separately measured by using a Y-shaped channel and a stenosed channel, respectively. Main improvement of this method is that blood sample can be accurately delivered into microfluidic device under *ex vivo* condition by adopting syringe pump into the rat extracorporeal model. Based on this improvement, various measurements can be possible using relatively small sample volume. This monitoring system has several distinctive advantages. First, the microfluidic system can measure various biophysical properties including blood viscosity, RBC aggregation and platelet adhesion using small blood sample without any labeling of cells. In addition, biophysical properties are directly measured under near-physiological conditions because of the usage of whole blood in the extracorporeal conduit. Finally, this system can quickly and precisely measure various properties by simple manipulation of the three-way direction valve. The utilization of syringe pumps enables to accurately delivery blood samples into microfluidic devices.

## Results

### Measurement of blood viscosity based on estimation of pressure ratio

**Figure 2:**
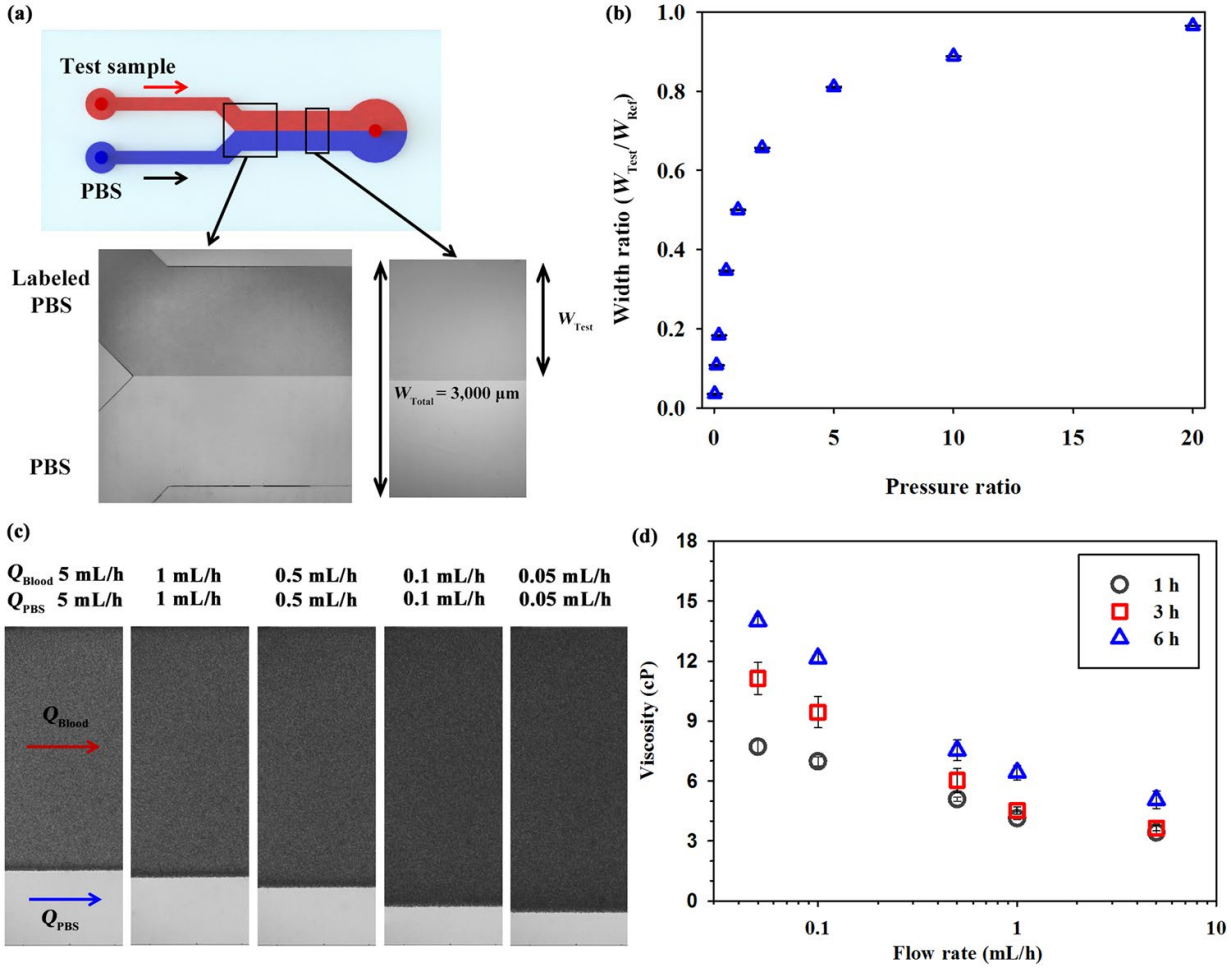
Measurement of variation in viscosity based on interfacial width between the test sample and PBS solution. (**a**) Schematic of the Y-shaped microfluidic device. An interfacial line is formed between the test sample and PBS solution by separately delivering the two fluids to each inlet of the microfluidic device. Optical images of the labeled PBS and PBS flows in the confluence region and interesting region. The channel downstream has a width (*W*_Total_) of 3000 μm and a height (*H*) of 50 μm. The width of the test fluid (*W*_Test_) is measured with image processing techniques. (**b**) Variation of the width ratio (*W*_Test_ */W*_Total_) according to pressure ratio between the labeled PBS and PBS solutions. After obtaining a fitting curve of the width ratio based on the regression analysis for the labeled PBS and PBS solutions, the pressure ratio between the two fluids is estimated from the width ratio curve. (**c**) Magnified images representing variations in the interfacial width of the blood samples (*W*_Test_) and PBS solution according to flow rate (*Q*_Blood_ and *Q*_PBS_ = 0.05 − 5 mL/h). (**d**) Variations in blood viscosity according to flow rate and lapsed time after LPS injection.

For viscosity measurement, the Y-shaped channel needs to estimate the ratio between a reference flow and the test blood flow. As shown in Fig. 2a, an interfacial line is clearly formed in the Y-shaped channel by separately delivering two different fluids. The interfacial width of the test sample is determined by the pressure difference between the two fluid flows. The relation between the interfacial width and pressure ratio between the two fluids is determined to measure viscosity of blood samples. Variations in the interfacial width according to pressure ratio are investigated by using reference PBS and PBS solution labeled with Rhodamine B dye at a low concentration (0.0125%). The reference PBS solution is delivered into the inlet of the Y-shaped channel at a constant flow rate of 5 mL/h, and the flow rate of the labeled PBS solution is varied in the range from 0.1 mL/h to 100 mL/h. Optical images in Fig. 2a show the flow conditions when the flow rates of the PBS and labeled PBS solutions are 5 mL/h. The width ratio indicates the ratio of the thickness of the labeled PBS solution (*W*_Test_) and total width of the downstream channel (*W*_Total_). The viscosities of the labeled PBS and PBS solution are almost the same, so the pressure ratio of labeled PBS to PBS (*P*_*Test*_ */P*_*Ref*_) is equal to their flow rate ratio (*Q*_*Test*_/*Q*_*Ref*_).

Figure 2b shows the variations in the width ratio according to pressure ratio. As the pressure ratio becomes higher, the width ratio also increases. The width ratio for labeled PBS dramatically increases with increasing pressure ratio by up to 5. After obtaining a fitting equation between the width ratio and pressure ratio, the pressure ratio between blood flow and PBS flow can be estimated by measuring the width ratio between them. Using the injection flow rates of the two fluid flows, unknown viscosity of blood sample is determined from the estimated pressure ratio. The detailed procedure of viscosity measurement is described in our previous study^27^.

Figure 2c illustrates the magnified images that represent the interfacial width between test blood samples and the reference PBS solution. For easy estimation of blood viscosity, the flow rates of blood sample and PBS solution are set to be the same. To obtain variations in blood viscosity according to shear rate, flow rates of blood and PBS are varied from 5 mL/h to 0.05 mL/h. As the flow rate of both fluids decreases, the width of blood samples increases. Supplementary Fig. s1a shows variations of viscosity of blood in rat samples without LPS treatment at 1, 3 and 6 h after establishing the rat extracorporeal model. Among non-LPS injected groups measured at 1, 3, and 6 h after PBS injection, statistical differences are not observed. However, the increasing tendency of blood viscosity is accentuated with the lapse of time after LPS injection (Fig. 2d). Considering the fact that the blood viscosity is mainly affected by RBC aggregation and deformability, the increased RBCs aggregability in the septic model contributes to hyperviscosity after LPS injection^9^.

### Monitoring variations in RBC aggregation caused by LPS injection

**Figure 3:**
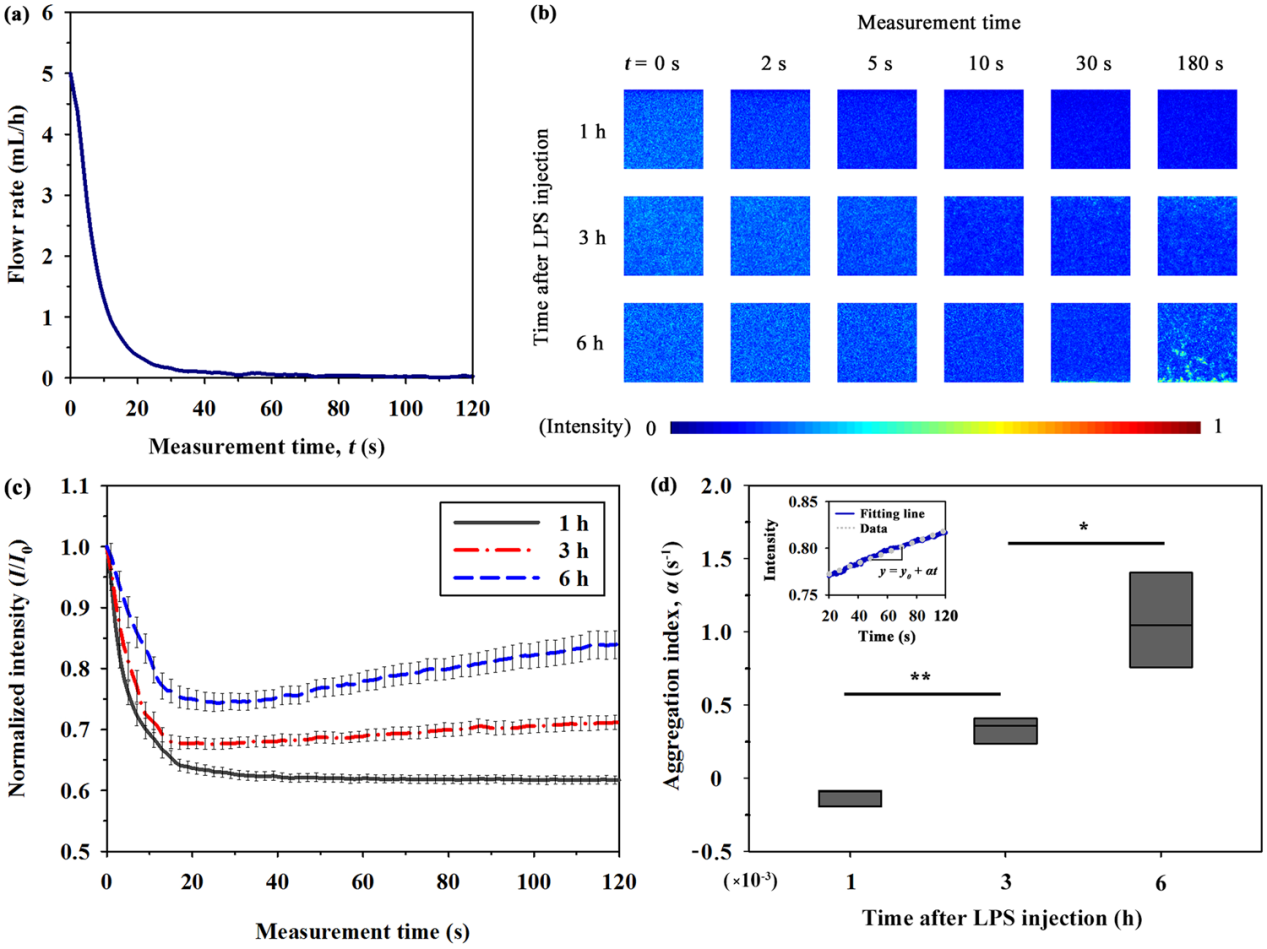
Variation in RBC aggregation measured by analyzing image intensity. (**a**) Temporal variation in flow rate of blood samples estimated by micro-PIV technique. (**b**) A series of speckle images for blood samples after the lapse of 1, 3, and 6 h from LPS injection with respect to measurement time. The size of each image is 128 × 128 pixels, corresponding to 0.64 × 0.64 mm^2^ in the physical dimension. (**c**) Temporal variations in the normalized light intensity (*I*/*I*_0_) of blood samples after 1, 3, and 6 h from LPS injection at the center of the channel. (**d**) Comparison of aggregation index (α) according to the measurement time after LPS injection. Light intensities of blood samples at 6 h after LPS injection, and the corresponding fitting curves are inserted. (^*^p < 0.005 and ^**^p < 0.001: significant difference between groups).

To induce the formation of RBC aggregates, the flow rate of blood samples is rapidly reduced by controlling the programmable syringe pump (5–0 mL/h). Considering that a certain discrepancy occurs between injection flow and real flow profiles because of the transient response of the microfluidic system, flow condition in the microchannel is monitored during the measurement of RBC aggregation. Figure 3a shows the temporal variation in flow rate evaluated based on velocity fields measured by a micro-particle image velocimetry (PIV). Details of the flow rate measurement are described in detail in our previous study^20^.

The optical images of blood flows are affected by the light transmitted through moving RBCs in the channel, so the formation of RBC aggregates alters the flow images. Figure 3b displays images of blood sample with respect to the measured elapsed time and time after LPS injection. The size of the region of interest (ROI) is 128 pixels, corresponding to 0.64 mm in physical dimension. RBC aggregates caused by the decrease in the shear rate, give rise to form speckle patterns accompanied with the increase in image intensity. For the blood samples tested after 3 and 6 h from LPS injection, blood images become brighter as the measurement time increases. However, variations are not so evident for blood samples tested after 1 h from LPS injection.

For detailed quantitative analysis, the light intensity (*I*) of images transmitted through the channel is normalized by the initial intensity value (*I*_0_) measured at the initial time of 0 s. Figure 3c shows temporal variations in normalized intensity (*I*/*I*_0_) with respect to the measurement time after LPS injection. Blood samples in the rat extracorporeal model have a slightly different hematocrit (Table 1). Taking into account the fact that the transmitted light intensity and speckle pattern in the microchannel are dependent on the hematocrit of blood samples^20^, the detailed variations in the normalized intensity are different for blood samples in the same group (same time after LPS injection). However, the hematocrit differences among the same groups do not induce a significant difference in the general trend of RBC aggregation. All of the normalized intensities are decreased in the initial stage (0–20 s). This decreasing trend is mainly caused by the shift in the Doppler frequency of photons scattered from the flowing RBCs^37, 38^. In particular, the decrease in flow rate reduces the intensity of light scattered from RBCs and the reduction of light leads to the decrease in the normalized intensity during the initial state. As the time after LPS injection increases, the decrement at the initial stage is gradually reduced because of enhanced RBC aggregation. Beyond the measurement time of 20 s, variation trends are somewhat different among groups The increment of normalized intensity is the largest for sample after 6 h from LPS injection (approximately 12% increase from 20 s to 120 s). By contrast, normalized light intensity values for samples after 1 h from LPS injection is reduced (approximately 3% decrease).

These variation trends are quantified as aggregation index (*α*), which represents the slope obtained by fitting the normalized light intensity values after a measurement time of 20 s. As a typical example, variations in normalized intensity of blood sample after 6 h from LPS injection and its fitting curve (*y* = *y*_0_ + *α*[*t* − 20]) are depicted in the inset of Fig. 3d. As shown in Fig. 3d, the aggregation index becomes higher as the time after LPS injection increases. Given that the formation of RBC aggregates induces brighter speckle patterns^20^, a high aggregation index implies that blood samples at a low flow rate condition can create speckle patterns continuously because of high aggregability. This variation trend is significantly different from the result for non-LPS injected samples (Supplementary Fig. s1b). Hyperaggregation of RBCs for LPS injected group may be related with the variation of plasma levels of molecules (fibrinogen and globulins) in response to the LPS treatment^12^. Following depletion theory, the exclusion of macromolecules (long-chain macromolecules including fibrinogen and globulins) between the RBCs leads to reduction of osmotic force in the vicinity of RBCs^39^. The aggregating force between RBCs is induced by the different osmotic pressure between the gap between RBCs, and bulk plasma^39, 40^.

### Estimation of platelet adhesion after LPS injection

**Figure 4:**
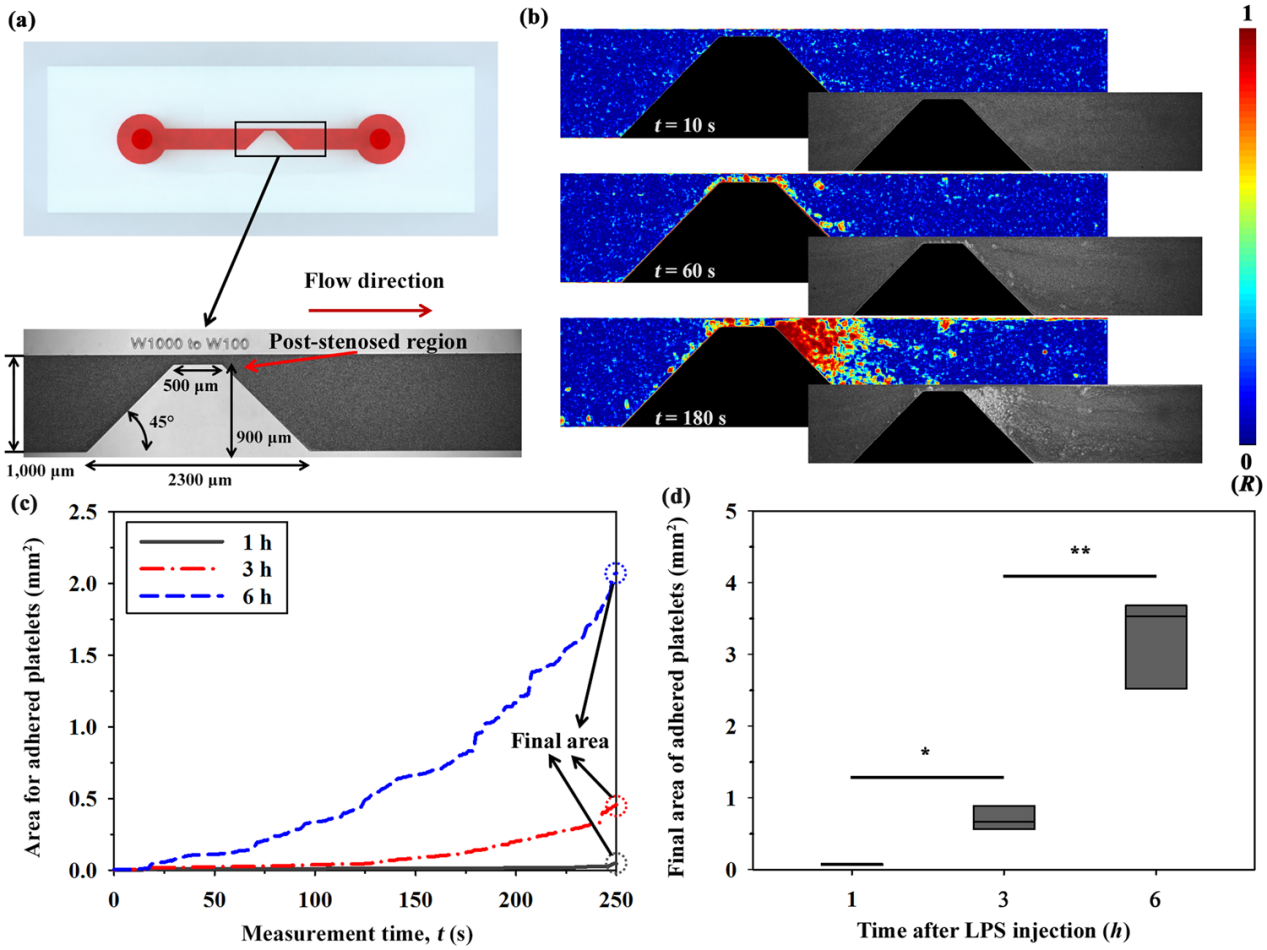
Estimation of platelet adhesion using a stenosed microchannel. (**a**) Schematic and optical image of the region of interest (ROI) of the stenosed microchannel. The severity of the stenosed channel is 90%. (**b**) Correlation maps depicting 2D correlation coefficient (*R*) in ROI after delivering blood samples at 6 h from LPS injection into the stenosed microchannel. Corresponding optical images are included for comparison. (**c**) Temporal variations in the area of the adhered platelet aggregates according to the lapsed time after LPS injection. (**d**) Comparison of the final area of adhered platelets at 250 s according to the lapsed time after LPS injection. (^*^p < 0.005 and ^**^p < 0.001: significant difference between groups).

For quantitative analysis of platelet activation, a microfluidic channel with severe stenosis is inserted in the rat extracorporeal conduit (Fig. 4a). By using a syringe pump, blood sample is supplied into the stenosed channel at a flow rate of 1 mL/h. Platelets passing through the narrow conduit are activated by shear acceleration. Given that the microchannel is coated with collagen, the activated platelets are adhered in the region just behind stenosed channel. Figure 4b shows the process of platelets adhesion with the lapse of time for a sample after 6 h from LPS injection. To accurately distinguish the adhered platelets from flowing blood, correlation maps of original images are utilized. Platelets are attached around the wall region of stenosis. After the initial adhesion of platelets, the size of the attached platelet aggregates increases with the lapse of time because of the active interaction between the platelets adhered to the collagen surface and flowing blood cells.

Figure 4c shows temporal variations in the area for adhered platelets. After the initial adhesion of platelets, the area for adhered platelets is slightly widened. Instants of the initial platelet adhesion and variation trend of the area for adhered platelets are significantly different among samples, so representative variations are depicted in Fig. 4c. As the lapse of time from LPS injection increases, platelets are easily adhered to the channel in the post-stenosed region. The area of adhered platelets is almost constant during measurement for samples at 1 h after LPS injection (increment of 0.0469 mm^2^ during the measurement). However, the area for the blood sample after 6 h from LPS injection exponentially increases with the increase in the measurement time. The area for adhered platelets at 250 s is defined as the final area and used as the representative parameter to compare platelet adhesion among groups. Although the final area of adhered platelets for non-LPS injected samples is slightly increased after establishing the rat extracorporeal model due to tissue factor caused by injured tissues caused by surgical treatment (Supplementary Fig. s1c), the mean values of final areas for a sample after 3 and 6 h from LPS injection increase about 9 and 43 times compared to the mean value for a sample after 1 h from LPS injection (Fig. 4d). This result may be attributed to variation of blood molecules and platelet activation caused by LPS inflammation^41, 42^.

### Temporal variation in conventional biomarkers

**Figure 5:**
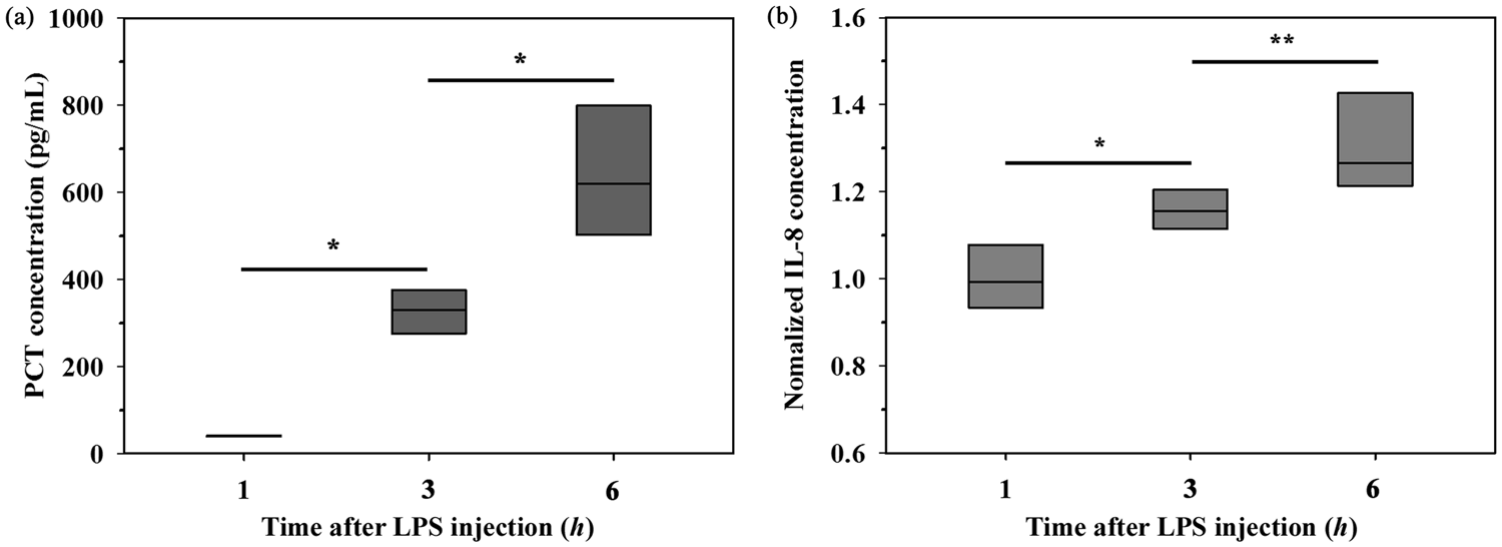
Variation in biomarkers for PCT and IL-8 extracted from a rat extracorporeal model. Comparison of biomarkers (**a**) PCT and (**b**) IL-8 in the plasma according to the lapsed time after LPS injection. (^*^p < 0.01 and ^**^p < 0.005: significant difference between groups).

In the rat extracorporeal model, sepsis is induced by LPS injection. In order to determine the presence or absence of sepsis in this model, PCT and IL-8, which are widely used as biomarkers, are measured according to the lapse of time after LPS injection (Fig. 5). The untreated whole blood samples (before LPS injection) have no detectable PCT in the plasma. The PCT concentration becomes measurable after 3 h from LPS injection. Similar to other biophysical properties, the PCT concentration increases with increasing elapsed time after LPS injection (about 7 and 15 times increase for a sample after 3 and 6 h from LPS injection compared to the mean value for a sample after 1 h from LPS injection). In contrast to PCT, the IL-8 concentration exhibits somewhat different values among various group samples. To obtain a general trend of the increment of the IL-8 concentration, the IL-8 concentration is normalized by dividing it with the initial IL-8 concentration prior to LPS injection. As expected, the normalized IL-8 concentration increases as the lapse of time after LPS injection increases. This result is consistent with that of previous studies, since the levels of these biomarkers are elevated with the lapse of time after septic infection^43, 44^.

## Discussion

The dose of LPS required to induce similar severity of sepsis in laboratory animals is much higher than that in humans (single dose of approximately 2 ng/kg LPS can produce profound physiologic effects in humans), because the response to endotoxin in rodent models is relatively more resistant compared with that in humans^34^. The inflammatory response of sepsis is induced through high-dose injection (3 mg/kg) of LPS into the rat extracorporeal model. Under *ex vivo* condition, the microfluidic system is found to reasonably monitor temporal variations in blood viscosity, RBC aggregation, and platelet adhesion because of inflammation in sepsis.

Bolus injection of LPS results in a highly rapid and transient increase in systemic cytokine levels and peripheral vascular resistance^45^. Cytokines are endogenous polypeptides or glycoproteins induced by tumour necrosis factor (TNF). Alterations in pro-inflammatory cytokines, including TNF-α, IL-1β, IL-6, and IL-8, play essential roles in the progress of septic shock or the failure of multiple organs^46^. PCT, which is a precursor of the calcitonin hormone released by bacteremia, has been suggested as a reliable biomarker in predicting sepsis^47^. To evaluate the degree of sepsis in the rat extracorporeal model, the concentrations of PCT and IL-8 in the plasma are measured. As time goes by after LPS injection, the concentrations of PCT and IL-8 become clearly noticeable (Fig. 5). Previous studies reported that the high level of PCT and IL-8 can indicate the degree of sepsis^7, 48^. In the present study, we checked that the degree of sepsis is pronounced with the lapse of time after LPS injection in the rat model. Based on this validation, it is reasonable to infer that variations in hemorheological properties and platelet adhesion are mainly caused by change in septic degree from mild sepsis to severe sepsis.

Given that platelets accumulated in the tissues of septic models contribute to organ damage^14, 49^, platelet activation may be associated to some extent with infectious states of sepsis. Although the detailed mechanism is still not fully established^14^, it may be closely related with the profound changes in microcirculation, cell adhesion and vascular leakage caused by sepsis infection^50^. Dysfunction of microcirculation is aggravated by alterations in hemorheological properties, such as reduced RBC deformability, increased RBCs aggregability, and enhanced adhesiveness among WBCs, platelets, and endothelial cells in animals and patients with sepsis^9, 12^. LPS and cytokines promote the production of nitric oxide (NO) and reactive oxygen species (ROS). As a modulator of the membrane properties of RBCs, NO and ROS decrease RBC deformability by increasing free intracellular Ca^2+^ ^51^. In addition, the reduced ATP levels of intracellular RBCs in patients with sepsis decrease the deformability of cells^52^. The increase in fibrinogen because of sepsis contributes to enhancement of RBC aggregation and blood viscosity^12^.

As shown in Figs 2–4, the increasing trends of blood viscosity, RBC aggregation, and platelet adhesion caused by sepsis are reasonably detected by the present microfluidic system. Considering the relationship between biophysical properties measured by the present system and the severity of sepsis, the proposed indices (aggregation index and final platelet area) may provide some biophysical information related to the inflammatory response of sepsis.

However, this system also has limitations. Above all, three different experiments should be conducted with changing microfluidic devices. For clinical applications, the measurement modalities of the present system should be integrated into one device. Since blood samples are collected from rodent models through exsanguination, a series of the same experiments using rat blood samples is commonly carried out for long-term treatment. Unlike usual cases, the present system can measure biophysical properties with relatively small sample volume (2.5 mL). This advantage allows monitoring temporal variations in biophysical properties after LPS injection of LPS in a short-term period (until 6 h). However, the continued consumption of blood sample restricts longitudinal analyses of biophysical parameters. For longitudinal analyses using the present microfluidic system, large-size animal models (e.g., pig or dog) have to be used. From this application, this system would be useful for a better understanding of the relationship between sepsis and biophysical parameters.

## Materials

### Fabrication of microfluidic device

A PDMS microfluidic device with depth of 50μm is fabricated by using soft lithography and deep reactive-ion etching. The Y-shaped microfluidic device has downstream channels with width (*W*_Total_) of 3000 μm and length of 11 mm. A stenosed channel has a contraction–expansion angle of 45° and severity of 90% (from 1,000 μm to 100 μm). PDMS prepolymer (Sylgard 184; Dow Corning, USA) is mixed with a curing reagent at a mass ratio of 10:1. The mixture is poured onto silicon molds. Air bubbles trapped in PDMS are removed in a vacuum chamber for 1 h. Subsequently, PDMS is cured at 80 °C for 3 h. The PDMS block is peeled off from the silicon molds. The inlet and outlets of the channels are made by a puncher of 1 mm diameter. After oxygen-plasma treatment (CUTE, Femto Science, Korea) of 40 W at a flow rate of 30 sccm under the base pressure of 0.923 Torr for 90 s, the microfluidic device is finally prepared by bonding the PDMS block to a glass substrate.

To facilitate platelet adhesion, the glass substrate is coated with collagen and the flow acceleration is induced in the stenosed channel^53^. The widths of the upstreamchannel and the stenosed apex are 1,000 μm and 100 μm, respectively. The severity of stenosis, which is defined as the ratio of stenosed apex area to the total cross-sectional area at upstreamchannel, is 90%. Shear rate in a rectangular channel can be calculated as^54^

1 $$\dot{\gamma }=(\frac{6Q}{W{H}^{2}})(1+\frac{H}{W}){f}^{\ast }(\frac{H}{W})$$

Here, *W* and *H* are the width and depth of the rectangular channel. *Q* is the flow rate and *f* ^*^ is a geometric constant depending on *H*/*W*. When *H*/*W* is 0.05 and 0.5 (aspect ratio at the upstreamchannel and the stenosed apex), the geometric constants of *f* ^*^ are 0.9365 and 0.6478, respectively. At a flow rate of 1 mL/h, shear rate at the upstreamchannel and stenosed apex are 650 and 6500 s^−1^, respectively. In a previous study, it was reported that wall shear rate over 300 s^−1^ can induce platelet adhesion on collagen thin films^55^. The shear rate conditions in this study are sufficient to monitor platelet adhesion.

### Preparation of septic rat samples

Rat samples (14 weeks old; n = 5 in each group) are anesthetized via intramuscular injection of ketamine (100 mg/kg) and xylazine (10 mg/kg). Prior to cannulation, an exact amount of heparin (1500 IU/mL/kg) is injected into the tail vein for anticoagulation. PE-50 tubes (ID = 0.58 mm, polyethylene tube) are cannulated into the left femoral artery and the right jugular vein of the rat samples. As shown in Fig. 1, a three-way tube with a valve connects the external conduit (PE-50 with inner diameter of 0.58 mm) to the microfluidic device and the 3 mL syringe in a syringe pump (neMESYS, Centoni Gmbh, Germany). By manipulating the three-way valve, blood samples are stably supplied to the microfluidic device by the syringe pump. PBS (pH 7.4, Bio Solution, Korea) solution is supplied into the other inlet of the channel. The mixture of the test blood and PBS solution is eliminated as waste. After establishing the rat extracorporeal model, 3 mg/kg of LPS (Escherichia coli O111:B4, Sigma-Aldrich) is injected into rat vein. The same measurement is repeated after 1, 3, and 6 h from LPS injection. To check the variation trend of biophysical properties for normal case, experimental data are obtained from non-LPS injected animals. Similar to the experiments for septic samples, blood viscosity, RBC aggregation index and area of adhered platelets are measured at 1, 3 and 6 hours after injecting 1 mL PBS into the rat extracorporeal model. The Animal Care and Ethics Committee of POSTECH has approved all the procedures performed on the test animals. All of the experiments are carried out in accordance with the approved guidelines.

### Experimental setup

The microfluidic devices connected to the extracorporeal conduit are placed on an optical microscope (Nikon, Tokyo, Japan) with 4x objective lens (NA of 0.1). Flow images in the microfluidic devices are consecutively captured with a high-speed camera (FASTCAM SA 1.1, Photron Ltd., San Diego, CA, USA) at a frame rate of 5000 fps. For the cases of Experiments II and III, six images are acquired when a trigger signal is generated from a delay generator (model 555, BNC, USA). The time interval between two trigger signals is 1 s. In the Experiment II, a micro-PIV technique is employed to measure velocity field of the flow using the cross-correlation PIV algorithm to each image pair. The size of each interrogation window is 32 × 32 pixels with 50% overlapping. The obtained velocity fields are filtered using a 3 × 3 median kernel. The detailed procedures of image acquisition and micro-PIV measurement are described in our previous study^20^.

### Image processing techniques for estimating platelet adhesion

To easily distinguish the adhered platelets from flowing blood in the captured optical images, the correlation mapping method is adopted. Each image is divided into 11 × 11 small tiles. The 2D correlation coefficient *R* of two tiles is calculated by using the following equation:

2 $$R=\frac{{\sum }_{i,j}(A(i,j)-\overline{A})(B(i,j)-\overline{B})}{\sqrt{({\sum }_{i,j}{(A(i,j)-\overline{A})}^{2})({\sum }_{i,j}{(B(i,j)-\overline{B})}^{2})}}\,$$

where, *A* and *B* represent the tiles of two consecutive images, (*i, j*) is the pixel coordinate of the tile, and $\overline{A}$ and $\overline{B}$ denote the mean values of the tiles *A* and *B*, respectively. After labeling *R* at a specific pixel, the labeling process is repeated for all captured images. The correlation maps are converted into binary images by adopting a thresholding method with an optimal value determined by Otsu’s algorithm. The area of adhered platelets is estimated by counting the number of nonzero pixels in the binary image. Given that the stenosed ROI has nonzero pixels, the stenosis region is masked prior to the calculation of the platelet area.

### PCT and IL-8 measurements

Plasma is isolated by centrifuging blood sample collected from the rat extracorporeal model. The concentrations of PCT or IL-8 in plasma are measured by ELISA kits (MyBioSource) following the manufacturer’s protocol. In brief, the isolated plasma is added on an antibody-coated 96 well plate to capture PCT or IL-8 in plasma. Detection antibody, horseradish peroxidase (HRP) conjugate, 3,3′,5,5′-tetramethylbenzidine (TMB) solution and stop solution are sequentially added in the plate with a washing process between each step. The absorbance of test samples is measured using a microplate reader (SPECTROstar) and analyzed using MARS Data Analysis Software (BMG Labtech).

### Statistical analysis

All data were expressed as mean value ± standard error of the mean (five samples for LPS treated group and three samples for non-LPS treated group). Differences between the two groups were compared using a two sample t test using commercial software (Matlab, Mathworks, USA).

## Electronic supplementary material

Supplementary information
