# Supplementary material for: Microfluidic system for monitoring temporal variations of hemorheological properties and platelet adhesion in LPS-injected rats
Source: Sci Rep. 2017 May 11;7:1801. doi: 10.1038/s41598-017-01985-w (PMC5431819; doi:10.1038/s41598-017-01985-w)
Supplement: Supplementary file 1 — Supplementary information [file 41598_2017_1985_MOESM1_ESM.pdf]

Microfluidic system for monitoring temporal variations of hemorheological properties and platelet adhesion in LPS-injected rats

Eunseop Yeom[1], Hye Mi Kim[2], Jun Hong Park[3], Woorak Choi[3],

Junsang Doh[2] and Sang Joon Lee[3]*

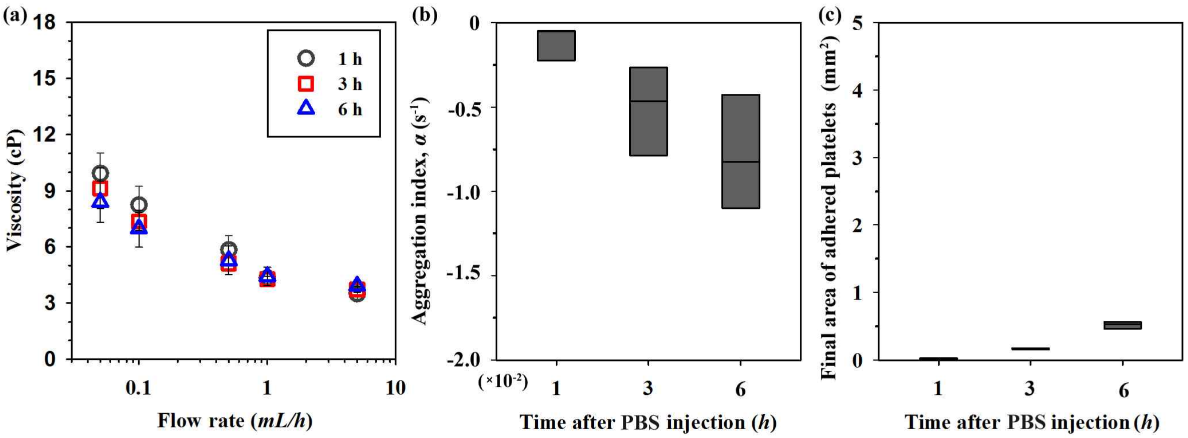

Supplementary Fig. s1 Temporal variations of biophysical properties for non-LPS injection group. **(a)** Variations of blood viscosity according to flow rate at 1, 3 and 6 h after injecting PBS into the rat extracorporeal model. Comparison of **(b)** aggregation index (α) and **(c)** final area of adhered platelets at 250 s after PBS injection (1, 3, and 6 h). Decrease in blood viscosity and RBC aggregability with the lapse of time may be related with reduced concentration of heparin in blood.
